# Costs Related to Frontotemporal Dementia in Latin America: A Scoping Review of Economic Health Studies

**DOI:** 10.3389/fneur.2021.684850

**Published:** 2021-08-23

**Authors:** Carlos Alva-Dìaz, Marco Malaga, Aaron Rodriguez-Calienes, Cristian Morán-Mariños, Victor Velásquez-Rimachi, Nilton Custodio

**Affiliations:** ^1^Grupo de Investigación Neurociencia, Efectividad Clínica y Salud Pública, Universidad Científica del Sur, Lima, Peru; ^2^Grupo Estudiantil de Investigación en Neurociencias, Sociedad de Estudiantes de Medicina Humana de la Universidad de San Martin de Porres, Lima, Peru; ^3^Facultad de Medicina Humana, Universidad de San Martín de Porres, Lima, Peru; ^4^Red de Eficacia Clínica y Sanitaria, Lima, Peru; ^5^Unidad de Investigación en Bibliometría, Universidad San Ignacio de Loyola, Lima, Peru; ^6^Unidad de Diagnóstico de Deterioro Cognitivo y Prevención de Demencia, Instituto Peruano de Neurociencias, Lima, Peru

**Keywords:** dementia, frontotemporal dementia, costs, cost analysis, Latin America

## Abstract

**Introduction:** Frontotemporal dementia (FTD) is a complex syndrome characterized by changes in behavior, language, executive control, and motor symptoms. Its annual economic burden per patient in developed countries has been classified as considerable, amounting to US$119,654 per patient, almost double the patient costs reported for Alzheimer's disease. However, there is little information regarding cost-of-illness (COI) for FTD in Latin-America (LA).

**Aim:** To describe the costs related to FTD in LA.

**Methods:** We included COI studies on FTD conducted in LA published in English, Spanish, or Portuguese from inception to September 2020. We carried out a systematic search in Pubmed/Medline, Scopus, Web of Science, Scielo, Cochrane, and gray literature. For quality assessment, we used a COI assessment tool available in the literature. All costs were reported in USD for 1 year and adjusted for inflation.

**Results:** We included three studies from Argentina, Brazil, and Peru. Direct costs (DCs) included medication (from US$959.20 to US$ 4,279.20), health care costs (from US$ 2,275.80 to US$7,856.16), and caregiver costs (from US$9,634.00 to US$28,730.28). Indirect costs (ICs) amounted to US$43,076.88.

**Conclusions:** In LA countries, the reporting of costs related to FTD continues to be oriented toward DCs. They remain lower than in developed countries, possibly due to the limited health budget allocated. Only one Brazilian report analyzed ICs, representing the highest percentage of the total costs. Therefore, studies on the COI of this disease in LA are essential, focusing on both out-of-pocket spending and the potential economic loss to patients' homes and families.

## Introduction

Frontotemporal dementia (FTD) is a complex syndrome characterized by clinical disorders that include progressive changes in the functions of behavior, language, executive control, and motor symptoms associated with anterior and frontal temporal lobe degeneration (1, 2). This dementia includes three different clinical phenotypes: the behavioral variant of primary progressive aphasia, the semantic variant, and the non-fluent/agrammatic variant of primary progressive aphasia; the most common being the behavioral variant of FTD (3).

Worldwide, FTD represents up to 5–6% of all dementias. Although less frequent than Alzheimer's disease (AD) (2, 4, 5), it is the second most common dementia in people under 65. FTD has an incidence of 1.61/100,000 and a mortality of 1.56/100,000 person-years (6). It is considered as early-onset dementia since it can present with an incidence of up to 10.8/100,000 people with a peak between 65 and 69 years of age (2, 7). In Latin American (LA) countries, FTD prevalence rates of 1.2–1.8/1,000 people have been described in populations over 60 years of age in Venezuela, Peru, and Brazil (8).

According to the World Health Organization (WHO), in 2015 the societal cost of dementia worldwide was US$818 billion, being equivalent to 1.1% of the global gross domestic product. Consequently, some reports classify the annual economic burden per FTD patient as considerable, amounting to US$119,654 per patient, being almost double the patient costs reported for AD (9). In developed countries, in which the annual income is high, these expenses are related to productivity, since the annual income of a patient can be reduced by up to US$ 50,000 at 12 months after diagnosis due to lost workdays and early layoffs (9). In LA countries, the expected socioeconomic impact of FTD is much more significant since the health budget allocated to this type of disease is small and may represent only 0.02% of the budget allocated to the health sector in low- and middle-income countries (9). Additionally, precarious labor systems assign low wages compared to those in developed countries, generating more significant uncertainty regarding the costs associated with FTD in the region.

The cost of illness (COI) is the value of resources spent or abandoned because of a health problem. It includes the costs of the health sector (direct costs [DCs]), the value of productivity diminished or lost by the patient (indirect costs [ICs]), and the cost of pain and suffering (intangible costs) (10, 11). DCs for the health sector include hospital expenses (hospitalization, treatment, and medical care) and, also, non-reimbursable expenses incurred by patients and family members concerning health care (medications, transportation for hospital visits, home modifications because of illness, and costs of caring for the patient at home). On the other hand, ICs can result from lost wages or benefits due to illness, premature death, side effects of illness or treatment, or time spent receiving treatment. ICs also affect family members who reduce or cease employment to care for the patient (10). There is an ongoing debate about whether caregiver costs represent DCs or ICs, as a caregiver can be formal or informal. However, there is growing consensus regarding the former (12, 13).

Although its calculation is complicated, COI analysis provides essential information on the financial impact of the disease in order to make more efficient use of resources (for example, select a specific treatment strategy) by health managers, researchers, and medical specialists. However, most studies on the economic burden of disease focus only on direct medical costs, as they are the easiest for the health sector to identify, and this underestimates the total costs (TCs) of the disease. Therefore, the objective in this systematic review was to describe the costs related to FTD in LA countries.

## Methods

We performed a scoping review to describe the costs related to FTD in LA countries with a protocol registered in the Figshare repository with 10.6084/m9.figshare.14100797 (14).

### Search Strategy

We carried out a systematic search in Pubmed/Medline, Scopus, Web of Science, Scielo, and Cochrane (OVID interface) from inception to September 2020. Additionally, we explored records of the Health Technology Assessment and assessed economic evaluation databases through https://www.crd.york.ac.uk/CRDWeb/. Finally, we carried out a manual search in the repositories of the WHO and the world bank.

We developed the search strategy from Medical Subject Headings–MeSH (Pubmed) for “Frontotemporal dementia” and related words for “Cost of the illness,” “Cost-Benefit Analysis,” and “Economics,” employing a PICO structure approach as much as possible as there was no intervention in our research question. Additionally, we developed a search filter for LA countries. The complete search strategy is shown in Supplementary Material 1.

### Selection of Studies

Two independent authors (MM & ARC) performed title and abstract screening, and ambiguities were discussed until consensus was reached. This was repeated for full-text selection, and a third author (CAD) participated if there was any discrepancy through discussion and consensus. Consensus was reached in every case.

We included studies conducted in LA countries published in English, Spanish or Portuguese. We considered studies of socioeconomic evaluation that provided data on the disease costs of FTD. Studies had to include a population diagnosed as FTD in accordance to the Lund and Manchester criteria (15). The aim of the study could either be to estimate COI, cost-effectiveness, cost-utility or cost-benefit. We excluded publication types such as letters, notes, conference papers, short surveys, and clinical trials, as well as studies not available in full text.

### Data Extraction and Quality Assessment

Two authors (MM & ARC) carried out the data collection independently using a standardized form in Microsoft Excel. A third author (VVR) verified the quality of the data before analysis. Additionally, authors were contacted to request unreported data.

The data extracted was made up of antecedents: author, year of publication, country, number of patients included in the study and the analysis, data collection method, calculation of costs, and quality of the articles. We categorized the cost components into direct medical costs and ICs. The first was made up of medication costs, health care services, direct social care costs, and caregiver expenses, while ICs included loss of productivity (10, 11).

Finally, we adjusted the costs for inflation using the Consumer Price Index inflation calculator of the US Bureau of Labor Statistics for all costs reported in US dollars (US$) by similar studies (16). We performed this calculation according to the year the exchange rate took place to its January 2021 value. The studies identified reported disease costs in US$ for one annual quarter or 1 month. We present all costs for 1 year for consistency, assuming there were no seasonal variations in resource use. We performed a qualitative analysis, as it was impossible to perform a meta-analysis due to the heterogeneity of the studies included.

For quality assessment, we adapted a tool developed for COI studies designed initially for diabetes by Afroz et al. (16). This tool has 15 indicators, which can be no score (0 points), a partial score (0.5 points), or a total score (1 point), with a maximum obtainable score of 15. However, three items were specific to the disease used for its development and did not apply to our studies, and therefore, the maximum obtainable score was 12. Two researchers (MM & ARC) independently assessed the risk of bias for each outcome.

## Results

### Selection and Characteristics of the Studies Included

We identified 920 studies in the databases. We eliminated 145 duplicates and screened a total of 775 by title and abstract. Of these, we assessed 20 studies in the full-text phase and selected three studies from four records for qualitative synthesis (17–19).

One study was developed in a Brazilian population (19) and the others, in a Peruvian (17) and an Argentinian population (18). There was a total of 333 patients with dementia, 61 of whom had FTD. The studies collected data from clinical records and an interview in one study (17, 20), only an interview in one (18), and a semi-structured questionnaire in another (19). Custodio et al. (17) and Rojas et al. (18) performed a single-center, retrospective cohort with a 3-month follow-up, while Ferreti et al. (19) carried out a single-center cross-sectional study. Only Ferreti et al. used the Resource Utilization in Dementia instrument for data collection (19). The characteristics of these studies are shown in Table 1.

**Table 1:** Characteristics of cost-of-illness studies in frontotemporal dementia in Latin America.

**References**	**Country**	**Study design**	**Follow-up**	**Patient population**				**Data collectin**	**RUD Applied**
				**Setting**	**Center**	**N**	**FTD**		
Custodio et al. (17)	Peru	Retrospective cohort	3 months	Outpatient	Private	136	18	Records & interview	No
Rojas et al. (18)	Argentina	Retrospective cohort	3 months	Outpatient	Public	104	34	Interview	No
Ferreti et al. (19)	Brazil	Cross-sectional	1 month	Outpatient	Public	93	9	Questionnaire	Yes

*FTD, Frontotemporal Dementia; N, number; RUD, Resource Utilization in Dementia instrument*.

### Risk of Bias of the Studies Included

The median score was 9 (range 9–9.5). In the general domain, only one study specifically defined FTD and the criteria employed for its diagnosis. Additionally, two of the studies (18, 19) collected data based only on self-assessment (questionnaire or semi-structured interview), while only one used records from care providers (17, 20). The follow-up time varied. Two studies (17, 18) covered 3 months of follow-up, and the other study (19, 20) involved only 1 month.

Although the reported costs and their components were not similar among studies, they were included individually as they agreed with the study objective. Additionally, not all studies discussed limitations.

As FTD patients were a minority of patients in all of the studies, none presented a sample representative of the population. However, the cost calculation approach was adequate in all studies, using a bottom-up approach, meaning that costs were obtained at the service provider level. Moreover, all the studies reported deviation standards and means and adequately performed and described the statistical analyses. Two studies (17, 18) performed multivariate regression analyses, while one (19) compared categories using the Chi-square test and ANOVA.

### Cost of Illness Analysis

#### Direct Costs

Table 2 summarizes the findings of each study and the definitions used in Supplementary Table 1. In broad terms, DCs included medical and social costs. The first was composed of medication and health care services, while social costs included non-medical costs such as clothing, transportation, or diapers, depending on each study. Additionally, we included caregiver expenses as DCs in our analysis.

**Table 2:** Annual cost-of-illness based on studies on frontotemporal dementia in Latin America.

**Study**			**Custodio et al. (17) (*n* = 18)**	**Rojas et al. (18) (*n* = 34)**	**Ferreti et al. (19) (*n* = 9)**
Direct costs X(%)	Medical costs	Medication	$4,279.20	$959.20	$2,310.48
		Anti-dementia	$0.00	$177.48	–
		Anti-psychotic	$4,279.20	$539.32	–
		Health care	$2,275.80	$4,463.80	$7,856.16
		Subtotal	$6,555.00	$5,423.00	$10,166.64
	Social care costs		–	–	$7,582.92
	Caregiver costs		$3,079.00	$677.64	$10,980.72
		Total Direct Costs	$9,634.00	$6,100.64	$28,730.28
Indirect costs: Productivity loss			–	–	$43,076.88
Total Costs			$9,634.00	$6,100.64	$71,807.16

*–, Data not reported*.

The criteria to define medication costs in the studies of Custodio et al. (17) and Rojas et al. (18) were similar, namely disease-specific drugs prescribed by a physician. Ferreti et al. included any medication-related costs, which ranged from US$959.20 in Argentina to US$ 4,279.20 in Peru per year. In both of these studies, anti-psychotic drugs were reported as making up a higher share of total medication costs, while Ferreti et al. did not report costs for specific drugs (19). Meanwhile, health care costs mainly included physician visits, medical tests, and hospitalizations. Ferreti et al. (19) included the cost of insurance. The annual health care costs varied from US$ 2,275.80 in Peru to US$ 7,856.16 in Brazil.

Thus, medical costs, which had the most homogeneous definition across studies, amounted to a total of US$5,423.00, US$6,555.00, and US$10,166.64 per year per patient in Argentina, Peru, and Brazil, respectively (17–19).

Social care costs were not reported by Rojas et al. (18). Custodio et al. (17) only reported diaper consumption per day, being US$0.00 (mode). In Brazil, the mean cost was US$ 631.91, which included diapers, transportation, and clothing (19).

Caregiver costs were analyzed separately as the definition varied across studies. Ferreti et al. reported the cost of informal caregivers, calculated using the time spent in patient care and the minimum wage the caregiver would receive (19). Rojas et al. reported only the cost of formal caregivers (18), while Custodio et al. included both approaches (17). These costs ranged from US$ 677.64 in Argentina to US$ 10,980.72 in Brazil.

#### Indirect Costs

Only the Brazilian study by Ferreti et al. (19) described ICs, reporting the projected annual loss of productivity for all patients with dementia according to their stage, mild, moderate, or severe, at $13,468.8, $18,106.8, and $ 19,736.4 US dollars, respectively. However, for FTD, the mean value across all stages was reported as US$43,076.88 per year.

#### Total Costs

**Figure 1:**
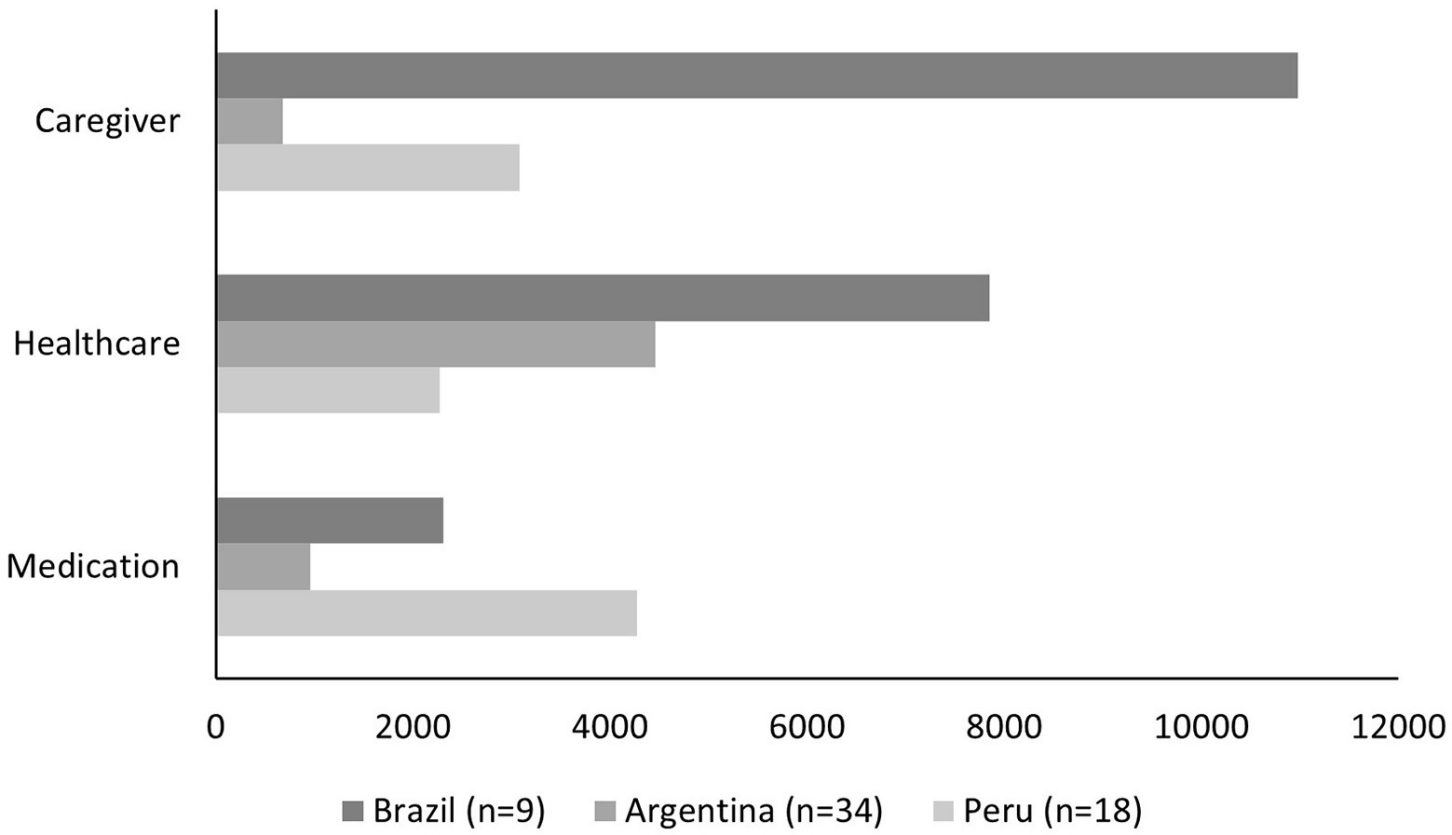
Frontotemporal dementia annual direct cost components in US Dollars for three Latin-American Countries.

Each study calculated TCs differently. In Brazil, Ferreti et al. (19) included medical costs, social care costs, caregiver expenses, and ICs (productivity loss), totaling up to US$71,807.16 per year. Meanwhile, Custodio did not include ICs and reported annual costs of US$9,634.00 (17). Finally, Rojas et al. did not account for either ICs or social care costs, reporting a total of US$6,100.64 per patient per year (18). Figure 1 shows a comparison among costs reported by the three studies.

Out-of-pocket expenses could not be quantitatively summarized for all studies, as the authors did not report them directly. Rojas et al. mentioned that all social-care costs were considered out-of-pocket expenses, but did not report them (18). However, the national health system covered all other costs. In Brazil, out-of-pocket expenses included both the medical costs not subsidized by the health care system and an undisclosed proportion of social-care costs (19). Finally, Custodio et al. conducted their study in a private health care center, including both insured and uninsured patients, but a breakdown in the costs between the two groups was not reported (17).

## Discussion

In this qualitative review, we describe the costs related to FTD in LA countries. We identified three studies from Peru, Argentina, and Brazil (17–19). In these moderate quality studies, TCs were reported in a range of $ 6,100.64 to $ 71,807.16 three studies and $ 43,076.88 in ICs in only one study.

The majority of LA studies focused their analysis on DCs related to medication and health service acquisition. These costs contrast to the experience in high-income countries, in which the focus of the ICs are on evaluating the economic impact by unemployment, decreased productivity, or individual-family care expenses (9). Most of the studies in LA countries did not report ICs, which could be due to the important difficulty in estimating these costs because of the lack of adequate data collection on labor productivity losses due to disease in LA countries. Despite this, in the study by Ferrati et al. (19), ICs were higher than in developing countries, similar to what is reported in high-income countries.

Medication expenses, from $ 5,423.00 to $ 10,166.64 annually, are still lower than those reported in countries with a higher development index, with DCs associated with annual dementia being from US$10,000 to US$60,000 (21–23). This difference may be due to the larger budget devoted to the health sector and subsidies in developed countries, providing better coverage for FTD patients than LA countries (24, 25). However, these expenses continue to be the most representative, corroborating their prominence and increase in LA as highlighted by the World Bank in 2019 (26).

Additionally, the reported caregiver costs ranged from $ 677.64 to $ 10,980.72. These DCs related to patient care are the highest, since the expenses related to the management of disability and dependency in FTD are as high as in other dementia conditions (27, 28). Therefore, the costs reported by caregivers and family members for the support of patients with FTD represent a significant percentage of the TCs identified in a similar way as in other reports, highlighting their negative economic impact on household income (29, 30). This percentage may be related to the special care and rehabilitation required by FTD patients to improve their functionality to provide well-being and better quality of life (31, 32). Consequently, this suggests that an important part of the costs are for expenses for the daily support of FTD patients. However, the economic impact of the loss of productivity of the patient and caregiver cannot be ignored, especially when the caregiver is a relative of the FTD patient, who could be prevented from working and being productive (29, 30). Policies in countries with socialized health care, in which the state can subsidize expenses related to outpatient care, including the assignment of a pension to health personnel or the relative in charge of home support, would greatly benefit patients in LA countries by reducing the individual expenses incurred by their families (33, 34).

Ferreti et al. (19) described out-of-pocket expenses as representing a large percentage of technical cooperation operations and TCs. Accurate identification of these costs is important because they represent the expenses that patients and families assume in their entirety, and therefore, represent the deterioration of the economic stability of the household. Unfortunately, quantifying these costs is very difficult due to the need for close surveillance to obtain reliable data. Reports indicate that out-of-pocket expenses in LA families may make up the central part of health spending, representing 10% to up to 60% of total spending, thereby being catastrophic expenses that can lead to and perpetuate family poverty (35, 36). COI studies must indicate out-of-pocket costs to propose public health measures that allow more adequate coverage of patients with FTD and their families in terms of medication and care expenses, reducing the significant economic impact on the families.

We critically evaluated the studies included using a COI-specific assessment tool (16). Overall, the quality of these studies was average, with heterogeneous data collection methods and small sample sizes, but they were transparent in cost descriptions and components and the statistical methods used. However, as all the studies were either retrospective or cross-sectional, there is a high risk of recall bias. Moreover, the follow-up period may have been too short to determine all the costs (from one to 12 months), and mainly ICs, which may be more clearly analyzed over more extended periods.

The main limitation of this review was the small number of patients included in the studies from only three countries, which affects the extrapolation of the results in the LA region. Furthermore, poor comparability due to different definitions and classifications of FTD patients produced poor comparability among studies. Additionally, the heterogeneity due to different data sources, different study design and different component of the costs should be considered. Therefore, the establishment of guidelines for COI studies in dementias other than AD would homogenize published information and future reviews. However, our report of the different types of costs was exhaustive. We adjusted the costs for inflation and for an annual period, which allows comparison of the costs reported in this work with other studies from different regions and countries.

## Conclusions

With moderate quality studies, we estimated a range of $ 6,100.64 to $ 71,807.16 in TCs and $ 43,076.88 in ICs. In LA countries, the reporting of costs related to FTD continues to be focused on DCs. These costs remain lower than in developed countries, possibly due to the limited health budgets allocated. Only one Brazilian report analyzed ICs, representing the highest percentage of the TCs. Therefore, studies on the COI of this disease in LA are essential and should be focused on both out-of-pocket spending and the potential economic losses to patients and families.

Expenditures should be appropriately distributed at public and individual health levels so that managers and specialists can provide efficient treatment options and well-being to patients with FTD. The knowledge gap related to indirect and intangible cost expenses in FTD creates an opportunity for interventions by interest groups in research and public managers.

## Data Availability Statement

The original contributions presented in the study are included in the article/Supplementary Material, further inquiries can be directed to the corresponding authors.

## Author Contributions

CA-D, MM, AR-C, CM-M, and VV-R planned and designed the protocol. NC and CA-D reviewed the methodological aspects of economic synthesis. VV-R, CM-M, MM, and AR-C wrote the manuscript. All authors designed the study and approved the final version of the manuscript.

## Conflict of Interest

The authors declare that the research was conducted in the absence of any commercial or financial relationships that could be construed as a potential conflict of interest.

## Publisher's Note

All claims expressed in this article are solely those of the authors and do not necessarily represent those of their affiliated organizations, or those of the publisher, the editors and the reviewers. Any product that may be evaluated in this article, or claim that may be made by its manufacturer, is not guaranteed or endorsed by the publisher.
