# Supplementary material for: Costs Related to Frontotemporal Dementia in Latin America: A Scoping Review of Economic Health Studies
Source: Front Neurol. 2021 Aug 23;12:684850. doi: 10.3389/fneur.2021.684850 (PMC8419437; doi:10.3389/fneur.2021.684850)
Supplement: Supplementary file 1 [file Data_Sheet_1.docx]

**Supplementary tables**

| Table S1. Cost-of-illness definitions given by studies in FTD in Latin-America | | | |
| --- | --- | --- | --- |
| Costs | Ferreti et al., 2015 | Rojas et al., 2010 | Custodio et al., 2015 |
| Direct costs | Medication, Healthcare, and Other Resources | Medical and Non-Medical | Direct Medical Costs and Direct Social Costs |
| Medical costs | - | Medical costs | Medical Costs |
| Medication | Market prices | Drugstore prices | Drugstore prices |
| Healthcare | Physician visits, health insurance | Hospital and nursing care, home care, services | Medical tests, medical visits, hospitalization |
| Social care costs | Other resources | Non-medical costs | Social costs |
|  | Diapers, transportation, clothing | Family out-of-pocket disease-related costs | Homecare, food supply, transport, residential or nursing home care |
| Caregiver | Informal caregiver (Care time) | Formal caregiver | Formal or informal caregiver |
| Indirect costs | Productivity loss (minimum wage) | - | Productivity loss (minimum wage) |

| Table S2. Quality assessment of cost-of-illness studies in Fronto-Temporal Dementia in Latin-America | | | | | |
| --- | --- | --- | --- | --- | --- |
| Dominion | Criteria | | Custodio, 2015 | Rojas, 2010 | Ferreti, 2015 |
| General | 1 | Is the definition clear and precise? | 0 | 1 | 0 |
|  | 2 | Which complications have the authors included? | - | - | - |
| Sample | 3 | Are sources for population data reliable? | 1 | 0.5 | 0.5 |
|  | 4 | The period of study is appropriate? | 1 | 1 | 1 |
| Costs | 5 | Does the study include the relevant costs? | 0.5 | 0.5 | 1 |
|  | 6 | Are the inclusion of the costs appropriate for the objective of the study? | 1 | 1 | 1 |
|  | 7 | Has the Diabetes severity Index been used? | - | - | - |
|  | 8 | Is adequate documentation and justification given for cost components, data, and sources, assumptions, and methods? | 1 | 1 | 1 |
|  | 9 | Are significant limitations discussed regarding the cost components, data, assumptions, and methods? | 1 | 1 | 1 |
| Methods | 10 | Is the data representative of the study population? | 0 | 0 | 0 |
|  | 11 | Was the approach appropriate? | 1 | 1 | 1 |
|  | 12 | Is the estimation method of the cost of diabetes appropriate? | - | - | - |
|  | 13 | Are the deviation standard and the means calculated? | 1 | 1 | 1 |
|  | 14 | Is a sensitivity analysis performed? | 0.5 | 0.5 | 0.5 |
|  | 15 | Which statistical methods are used? | 1 | 1 | 1 |
|  |  | Total | 9 | 9.5 | 9 |
| “-“: Not met the criteria. | | | | | |

# Supplementary Material 1.

PRISMA Flow Diagram

**Search strategy**

| Database |  | Results |
| --- | --- | --- |
| Scopus | #1 ( ( TITLE-ABS-KEY ( "frontotemporal dementia" ) OR TITLE-ABS-KEY ( dementia* W/2 frontotemporal ) OR TITLE-ABS-KEY ( dementia* W/1 semantic ) OR TITLE-ABS-KEY ( dementia* W/1 disinhibition ) OR TITLE-ABS-KEY ( atroph* W/1 lobar) ) OR ( TITLE-ABS-KEY ( "Multiple System Tauopathy" OR "Ubiquitin positive" OR "Hereditary Dysphasic Disinhibition Dementia" OR "FTDP-17" OR "TDP-43 Pathology" OR DDPAC or dementia*) ) ) OR ( ( TITLE-ABS-KEY ( disease* ) AND TITLE-ABS-KEY ( "wilhelm lynch" OR picks ) ) )  #2 (((TITLE-ABS-KEY(analys*) AND TITLE-ABS-KEY("Cost-Benefit" or "Cost- Utility" or marginal)) OR (TITLE-ABS-KEY(cost) AND TITLE-ABS-KEY(benefit* or Effectiveness)) OR TITLE-ABS-KEY(“Cost of illness” or Economic*)) OR (TITLE-ABS-KEY(economic*) AND TITLE-ABS-KEY(evaluation* or study)))  #3 ALL ( argentina OR bolivia OR brazil OR chile OR colombia OR "Costa Rica" OR cuba OR ecuador OR "El Salvador" OR guatemala OR haiti OR honduras OR mexico OR suriname OR nicaragua OR panama OR paraguay OR peru OR "Puerto Rico" OR "Dominican Republic" OR uruguay OR venezuela OR "Latin America" OR caribbean )  ((#1 AND #2) and #3) | 488 |
| Pubmed / Medline | #1 "frontotemporal dementia"[mh] or dementia[mh] or (dementia*[tiab] and frontotemporal[tiab]) or (ubiquitin[tiab] and positive[tiab]) or ("Multiple System Tauopathy"[tiab] OR "Ubiquitin positive"[tiab] or "Hereditary Dysphasic Disinhibition Dementia"[tiab] or "FTDP-17"[tiab] OR "TDP-43 Pathology"[tiab]) or (disease*[tiab] and ("Wilhelmsen Lynch"[tiab] or picks[tiab]))  #2 "Cost-Benefit Analysis"[Mh] or economics[mh] or (economic[tiab] and evaluation*[tiab]) or (Analys*[tiab] and (economic[tiab] or "Cost-Benefit"[tiab] or "Cost-Utility"[tiab] or marginal[tiab])) or (cost[tiab] and (benefit*[tiab] or Effectiveness[tiab])) or “Cost of illness”[tiab]  #3 Latin America[Mesh] OR Latinoamerica*[all] OR Hispanic Americans[Mesh] OR Panamerican*[all] OR Central America[Mesh] OR Centroamerica*[all] OR South America[Mesh] OR "South America"[all] OR Southamerica*[all] OR Sudamerica*[all] OR “America del sur”[all] OR Caribbean Region[Mesh] OR Amazon*[all] OR Argentin*[all] OR Bolivia*[all] OR Brazil*[all] OR Colombia*[all] OR Chile*[all] OR Chile[pl] OR Ecuador*[ad] OR Ecuador*[all] OR Guiana*[all] OR Guyana[all] OR Paraguay*[all] OR Peru*[all] OR Surinam*[all] OR Uruguay[all] OR Venez*[all] OR Costa Ric*[all] or Costaric*[all] OR Salvador*[all] or Guatemala[all] OR Hondur*[all] OR Nicaragu*[all] OR Panama[all] OR Mexico[Mesh] OR Mexic*[all] OR Cuba*[all] or Dominican Republic[all] OR Haiti[all] OR Jamaic*[all] OR Puerto Ric*[all]  ((#1 AND #2) and #3) | 107 |
| Web Of Science | #1 (TS=(dementia*) and TS=(frontotemporal or disinhibition or semantic)) or TS=(atroph* and lobar) or TS=("Multiple System Tauopathy" OR "Ubiquitin positive" OR "Hereditary Dysphasic Disinhibition Dementia" OR "FTDP-17" OR "TDP-43 Pathology" OR DDPAC or dementia*) or (TS=(disease*) and TS=("wilhelm lynch" OR picks))  #2 (TS=(analys*) and TS=("Cost-Benefit" or "Cost- Utility" or marginal)) or (TS=(cost) and TS=(benefit* or Effectivenes)) or (TS=(economic*) and TS=(evaluation* or study)) or TS=(“Cost of illness” or economic*)  #3 ALL=(argentina OR bolivia OR brazil OR chile OR colombia OR "Costa Rica" OR cuba OR ecuador OR "El Salvador" OR guatemala OR haiti OR honduras OR mexico OR suriname OR nicaragua OR panama OR paraguay OR peru OR "Puerto Rico" OR "Dominican Republic" OR uruguay OR venezuela OR "Latin America" OR caribbean)  ((#1 AND #2) and #3) | 128 |
| Scielo | #1 (TS=(dementia*) and TS=(frontotemporal or disinhibition or semantic)) or TS=(atroph* and lobar) or TS=("Multiple System Tauopathy" OR "Ubiquitin positive" OR "Hereditary Dysphasic Disinhibition Dementia" OR "FTDP-17" OR "TDP-43 Pathology" OR DDPAC or dementia*) or (TS=(disease*) and TS=("wilhelm lynch" OR picks))  #2 (TS=(analys*) and TS=("Cost-Benefit" or "Cost- Utility" or marginal)) or (TS=(cost) and TS=(benefit* or Effectivenes)) or (TS=(economic*) and TS=(evaluation* or study)) or TS=(“Cost of illness” or economic*)  #3 ALL=(argentina OR bolivia OR brazil OR chile OR colombia OR "Costa Rica" OR cuba OR ecuador OR "El Salvador" OR guatemala OR haiti OR honduras OR mexico OR suriname OR nicaragua OR panama OR paraguay OR peru OR "Puerto Rico" OR "Dominican Republic" OR uruguay OR venezuela OR "Latin America" OR caribbean)  ((#1 AND #2) and #3) | 58 |
| Cochrane (Ovid) | #1 ("frontotemporal dementia" or "Multiple System Tauopathy" or "Ubiquitin positive" or "Hereditary Dysphasic Disinhibition Dementia" or "FTDP-17" or dementia*).ab,kw,sh,ti,tw.  #2 ("Cost-Benefit Analysis" or "cost-benefit" or cost-utility or cost* or economic*).ab,kw,sh,ti,tw.  #3 (argentina or bolivia or brazil or chile or colombia or "Costa Rica" or cuba or ecuador or "El Salvador" or guatemala or haiti or honduras or mexico or suriname or nicaragua or panama or paraguay or peru or "Puerto Rico" or "Dominican Republic" or uruguay or venezuela or "Latin America" or caribbean).af.  ((#1 AND #2) and #3) | 139 |
| (Health Technology Assessment Database (HTA) / CRD assessed economic evaluation | #1 ((economic*):TI OR (cost*):TI AND (dementia*):TI) and ((Economic evaluation:ZDT and Bibliographic:ZPS) OR (Economic evaluation:ZDT and Abstract:ZPS) OR Project record:ZDT OR Full publication record:ZDT) IN NHSEED, HTA | 45 |

| **Studies** | **Reason for exclusion** |
| --- | --- |
| Allegri, R. F., et al. (2007). "Economic impact of dementia in developing countries: An evaluation of costs of Alzheimer-type dementia in Argentina." International Psychogeriatrics 19(4): 705-718. | Different population |
| Alva-Diaz, C., et al. (2020). "Neurological diseases in Peru: a systematic analysis of the global burden disease study." Arquivos de Neuro-Psiquiatria 78(5): 282-289. | Different objective |
| Bustin, J., et al. (2020). "What is happening with not recommended drugs for dementia in Argentina? Prescription patterns and direct costs analysis." International Journal of Geriatric Psychiatry 35(3): 270-275. | Different objective |
| Cabello, H. R., et al. (2012). "The economic impact of mental health services and the need for cost reduction programs: Suggestions from middle-income countries." Acta Psychiatrica Scandinavica 126(4): 298-299. | Different study design |
| Camargo, C. H. F., et al. (2015). "Patients with dementia syndrome in public and private services in southern Brazil." Dementia e Neuropsychologia 9(1): 64-70. | Different objective |
| Costa, N., et al. (2013). "Comparison of informal care time and costs in different age-related dementias: A review." BioMed Research International 2013. | Different population |
| Goldfeld, K. S., et al. (2013). "The cost-effectiveness of the decision to hospitalize nursing home residents with advanced dementia." Journal of Pain and Symptom Management 46(5): 640-651. | Different objective |
| Henderson, C., et al. (2019). "Use and costs of services and unpaid care for people with mild-to-moderate dementia: Baseline results from the IDEAL cohort study." Alzheimer's and Dementia: Translational Research and Clinical Interventions 5: 685-696. | Different population |
| König, H. H., et al. (2014). "The Costs of Dementia From the Societal Perspective: Is Care Provided in the Community Really Cheaper than Nursing Home Care?" Journal of the American Medical Directors Association 15(2): 117-126. | Different study design |
| Maestre, G. E. (2012). "Assessing dementia in resource-poor regions." Current Neurology and Neuroscience Reports 12(5): 511-519. | Different population |
| Prada, M. E. R., et al. (2017). "ECONOMIC IMPACT ON THE PATHOLOGIES OF MENTAL HEALTH, NEUROLOGICAL AND DEMENTIA IN COLOMBIA." Value in Health 20(9): A898-A898. | Different study design |
| Prince, M. J., et al. (2016). "The economic status of older people's households in urban and rural settings in Peru, Mexico and China: a 10/66 INDEP study cross-sectional survey." SpringerPlus 5. | Different objective |
| Schaller, S., et al. (2015). "The main cost drivers in dementia: A systematic review." International Journal of Geriatric Psychiatry 30(2): 111-129. | Different population |
| Veras, R. P., et al. (2007). "Family care for demented elderly individuals: Cost analysis." Revista de Psiquiatria Clinica 34(1): 5-12. | Different population |
| Hojman, Daniel A., et al. "The cost of dementia in an unequal country: the case of Chile." PLoS One 12.3 (2017): e0172204. | Different objective |
